# Species-dependent structural polymorphism of Y145Stop prion protein amyloid revealed by solid-state NMR spectroscopy

**DOI:** 10.1038/s41467-017-00794-z

**Published:** 2017-09-29

**Authors:** Theint Theint, Philippe S. Nadaud, Darryl Aucoin, Jonathan J. Helmus, Simon P. Pondaven, Krystyna Surewicz, Witold K. Surewicz, Christopher P. Jaroniec

**Affiliations:** 1 Department of Chemistry and Biochemistry, The Ohio State University, Columbus, OH 43210 USA; 2 Department of Physiology and Biophysics, Case Western Reserve University, Cleveland, OH 44106 USA

## Abstract

One of the most puzzling aspects of the prion diseases is the intricate relationship between prion strains and interspecies transmissibility barriers. Previously we have shown that certain fundamental aspects of mammalian prion propagation, including the strain phenomenon and species barriers, can be reproduced in vitro in seeded fibrillization of the Y145Stop prion protein variant. Here, we use solid-state nuclear magnetic resonance spectroscopy to gain atomic level insight into the structural differences between Y145Stop prion protein amyloids from three species: human, mouse, and Syrian hamster. Remarkably, we find that these structural differences are largely controlled by only two amino acids at positions 112 and 139, and that the same residues appear to be key to the emergence of structurally distinct amyloid strains within the same protein sequence. The role of these residues as conformational switches can be rationalized based on a model for human Y145Stop prion protein amyloid, providing a foundation for understanding cross-seeding specificity.

## Abstract (web summary)

Prion diseases can be transmitted across species. Here the authors use solid-state NMR to study prion protein (PrP) amyloids from human, mouse and Syrian hamster and show that their structural differences are mainly governed by two residues, which helps to understand interspecies PrP propagation on a molecular level.

## Introduction

Prion diseases are a diverse group of transmissible neurodegenerative disorders that affect both humans as well as many animal species^1–4^. All these diseases are associated with the misfolding and aggregation of the prion protein (PrP), a membrane associated glycoprotein of still unknown physiological function. The self-propagating conversion of the normal, monomeric form of PrP (denoted PrP^C^) to a polymeric, β-sheet-rich conformer (PrP^Sc^) is believed to be a critical molecular event in the pathogenesis of prion diseases^1–4^. Furthermore, a wealth of experimental data supports the “protein-only” model, which implies that PrP^Sc^ itself is the infectious prion agent^1–10^.

One of the most intriguing and still poorly understood aspects of prion diseases is the existence of multiple prion strains that give rise to different disease phenotypes within the same mammalian species^1–3^. In the context of the protein-only model, individual prion strains are believed to be encoded as structurally distinct PrP^Sc^ aggregates^1–3, 11–16^. However, despite recent progress^17, 18^, the nature of these structural differences is still poorly defined, largely due to fundamental technical difficulties of structural studies with brain-derived prions. Another confounding issue relates to mechanisms that control disease transmission barriers between different species. Growing number of data indicate that these barriers (or lack thereof) result from a complex interplay between species-dependent amino acid sequence differences in PrP and prion strain characteristics^3, 14, 19–23^. However, molecular level details of this interplay remain largely unknown.

In a series of studies, we have used amyloid fibrils formed by the Y145Stop PrP fragment (PrP23-144) as a model for studying molecular aspects of prion propagation^24–26^. This C-terminally truncated PrP variant is associated with one of the familial human prion diseases^27, 28^, and recent studies indicate that PrP23-144 fibrils are infectious, causing a transmissible neurodegenerative disease in mice^10^. Even though no similar C-terminal truncations of PrP have been reported in other species, this simple model allowed us to reproduce in vitro certain aspects of mammalian prion propagation, providing insight into the mechanism responsible for species-dependent and strain-dependent seeding barriers^25, 26^. One of the important findings was that the key determinant of seeding specificity is the ability of PrP monomer from a given species to adapt to the structure of a particular PrP23-144 amyloid strain. However, this mechanistic model is still largely conceptual in nature, lacking description at the atomic level.

The unique advantage of PrP23-144 amyloid fibrils is that, unlike other mammalian prions, this system is readily amenable to high-resolution structural analysis by solid-state nuclear magnetic resonance (NMR) spectroscopy^29–31^. Our previous structural studies focused primarily on human PrP23-144 fibrils^32–36^, revealing that the rigid β-sheet core of these fibrils consists of three relatively short β-strands within the region between residues 112–141, and that this β-core region displays a parallel in-register organization^32–34^. This overall motif of parallel in-register β-structure is common to amyloids formed in vitro by different PrP variants including PrP90-231^37^ and full-length protein^38, 39^, even though recent data indicate that the structure of at least some strains of brain-derived prions is based on the β-solenoid motif^40^. Here, we report solid-state NMR data that provide the initial high-resolution insights into the structural differences between PrP23-144 fibrils from three different species (human, mouse, and Syrian hamster) and identify specific amino acid residues that control these differences. Furthermore, our studies suggest that the same amino acid residues play critical roles in the emergence of structurally distinct strains of PrP23-144 amyloid fibrils within the same amino acid sequence. These structural insights provide a foundation for more complete understanding of the molecular basis of cross-seeding in prion propagation between species.

## Results

### Residues controlling PrP23-144 amyloid core structure

As shown previously, freshly dissolved PrP23-144 is monomeric and largely unstructured, but upon incubation it converts to β-sheet-rich amyloid fibrils^24^. In this section, we describe solid-state NMR studies of human, mouse, and Syrian hamster PrP23-144 amyloids (referred to as [hu], [mo], and [Sha], respectively), as well as amyloids formed by select single or double point mutants of these proteins, generated at 25 °C under quiescent conditions as described in the Methods section. Under these conditions all of the PrP23-144 variants studied assemble into micron length amyloid fibrils as documented in our previous studies using atomic force microscopy and thioflavin T fluorescence^24–26^. Furthermore, all of the fibril samples are reproducibly homogeneous on the molecular scale based on effectively identical fingerprint 2D ^15^N–^13^Cα solid-state NMR spectra recorded for multiple independent preparations (Supplementary Fig. 1 for representative data).

**Fig. 1:**
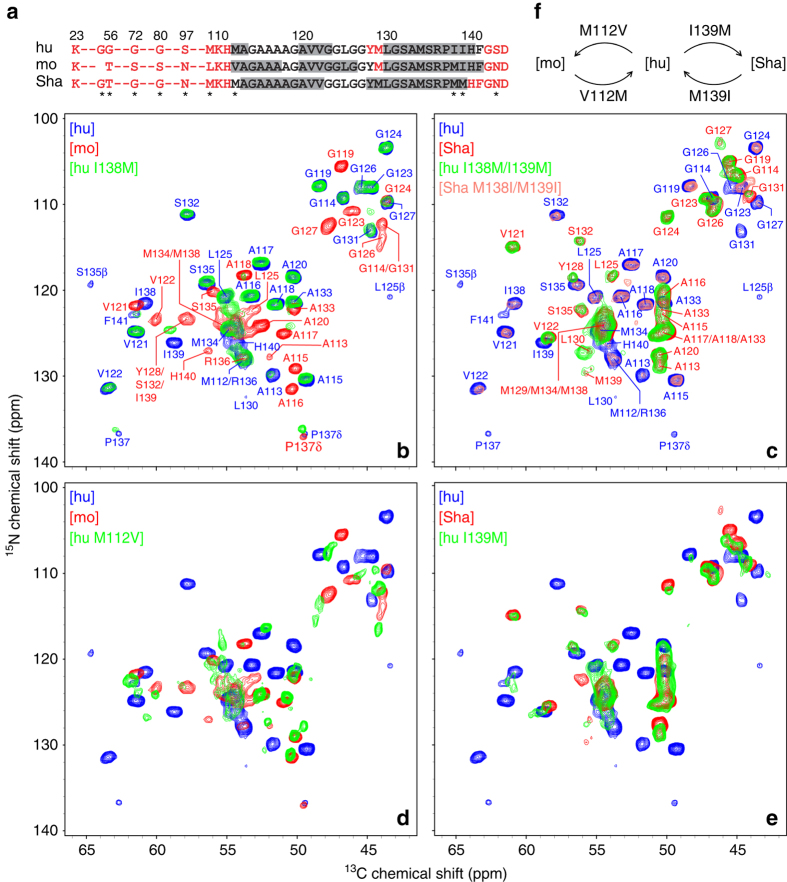
Two-dimensional ^15^N–^13^Cα solid-state NMR spectra of PrP23-144 amyloid fibrils. **a** Amino acid sequences of hu, mo, and ShaPrP23-144. Immobile residues, located within the amyloid β-core and observable in solid-state NMR spectra, are shown in *black font*, and undetectable, conformationally flexible residues are shown in *red font*. Residues having the highest β-strand propensity according to TALOS-N^41^ chemical-shift based secondary structure analysis described previously^36^ are indicated by *gray rectangles*. The asterisks indicate all residues that are not conserved between the hu, mo, and ShaPrP23-144 sequences. **b**–**e** Two-dimensional ^15^N–^13^Cα fingerprint NMR spectra of PrP23-144 amyloid fibrils recorded at 500 or 800 MHz ^1^H frequency, 11.111 kHz MAS rate, and 5 °C. Spectra are shown for a total of eight PrP23-144 amyloids, including the following proteins and their single or double mutants: [hu], [mo], [Sha], [hu M112V], [hu I138M], [hu I139M], [hu I138M/I139M], and [Sha M138I/M139I], as indicated in the insets and by contours of corresponding color. Also shown in corresponding color in **b** and **c** are the resonance assignments for [hu], [mo], and [Sha] fibrils^32, 36^. **f** Summary of the key amino acid substitutions that, based on the solid-state NMR data in this study, are primarily responsible for the PrP23-144 amyloid β-core adopting [hu], [mo], or [Sha]-like structures

The ^13^C and ^15^N chemical shift assignments for [mo] and [Sha] fibrils were established by using 2D and 3D solid-state NMR experiments as discussed in detail elsewhere^36^. Assigned 2D ^15^N–^13^Cα spectra for [mo] and [Sha] fibrils are shown in Fig. 1b, c, respectively, and reveal that, in close analogy to [hu] amyloid^32, 33^, the [mo] and [Sha] amyloids each contain a relatively rigid and compact C-terminal β-core region spanning ~30 amino acids (residues ~112–140) with the remainder of the protein dynamically disordered. The finding that the backbone chemical shifts differ markedly between the fibrils for all three species in spite of nearly identical amino acid sequences (^13^Cα chemical shift root mean squared deviation, δ(^13^Cα) RMSD, values for the common residues were found to be 1.9, 2.3, and 1.9 ppm for [hu] vs. [mo], [hu] vs. [Sha], and [mo] vs. [Sha], respectively^36^) indicates that hu, mo, and ShaPrP23-144 adopt three-dimensional amyloid β-core structures that are distinct from one another at the atomic level. The chemical shift based secondary structure analysis using TALOS-N^41^ summarized in Fig. 1a (see also Supplementary Fig. 2 for plots of ^13^Cα–^13^Cβ secondary chemical shift differences), points to particularly significant structural differences between the β-core regions of [hu] and [Sha] amyloids: three segments with especially high β-strand propensity, aa ~112–113, ~120–123, and ~130–140, for [hu] vs. two extended β-strands, aa ~113–122 and ~128–139, for [Sha]. On the other hand, in spite of the considerable chemical shift differences, [mo] amyloid appears to adopt a molecular conformation bearing some similarity to its [hu] counterpart, with three predicted β-strands spanning residues ~112–117, ~120–126, and ~130–141. It is also noteworthy that the [hu], [mo], and [Sha] amyloids all exhibit nearly identical fibril mass-per-lengths (MPLs), on the order of 50–55 kDa nm^−1^, according to analysis by tilted-beam transmission electron microscopy (TB-TEM)^42^ (Supplementary Fig. 3). For [hu] amyloid, which has been previously found to assemble into a parallel in-register β-sheet structure^34^, this MPL value corresponds to two PrP23-144 molecules per 0.47-nm β-sheet repeat spacing. Thus, the structural diversity between [hu], [mo], and [Sha] amyloids implied by the observed differences in NMR chemical shifts is not the result of large-scale disparities in fibril supramolecular architectures.

Previous studies employing biochemical and low-resolution biophysical techniques^25, 26^ have demonstrated that [hu], [mo], and [Sha] amyloids exhibit distinct secondary structures and morphologies and that these features are strongly correlated with the observed seeding specificities. Notably, these studies also identified species-dependent residues 138 and 139 (Ile-Ile, Met-Ile, and Met-Met for hu, mo, and ShaPrP23-144, respectively) as critical determinants of fibrillization kinetics, fibril morphology, and seeding specificity. To assess the influence of these amino acids on the atomic structure of PrP23-144 amyloid, we recorded 2D ^15^N–^13^Cα solid-state NMR spectra for several PrP23-144 variants targeting residues 138 and 139. Figure 1c shows that NMR spectra for amyloid from huPrP23-144 with hamster-like substitutions of Ile 138 and Ile 139 ([hu I138M/I139M]) and [Sha] amyloid are effectively identical (δ(^13^Cα) RMSD = 0.2 ppm), indicating that replacement of these two isoleucine residues in huPrP23-144 by methionines is sufficient to drive protein assembly into fibrils with a β-core structure that is characteristic of [Sha] rather than [hu] amyloid. To further validate this finding, we have generated [Sha M138I/M139I] fibrils, with Met 138 and Met 139 in ShaPrP23-144 replaced by isoleucines, and obtained ^15^N–^13^Cα NMR spectra (Fig. 1c) that mirror those for [hu] amyloid (δ(^13^Cα) RMSD = 0.1 ppm). In contrast, analogous experiments for huPrP23-144 with a single mouse PrP-like substitution of Ile 138 with Met surprisingly revealed that [hu I138M] amyloid adopts a [hu] rather than [mo]-like fold (Fig. 1b; δ(^13^Cα) RMSD = 0.1 ppm for [hu] vs. [hu I138M]). The latter result indicates that, in spite of the fact that the I138M mutation alters the fibrillization kinetics of huPrP23-144 to be nearly identical to those for moPrP23-144^25^, the exact identity of this residue is of little, if any, consequence for the final amyloid structure.

The unexpected finding that residue 138 is not a critical determinant of PrP23-144 amyloid structure immediately raises the following two questions: (i) Is the I139M substitution alone sufficient to induce huPrP23-144 into forming a [Sha]-like amyloid structure? and (ii) Which amino acid substitutions in huPrP23-144 are key for protein assembly into a [mo]-like amyloid structure?

The answer to the first question is clearly affirmative—that is, the I139M substitution indeed acts as a sole conformational switch between [hu] and [Sha]-like amyloid β-core structures. This assessment is based on the finding that the ^15^N–^13^Cα NMR spectrum for [hu I139M] fibrils (Fig. 1e) is effectively identical to the corresponding spectrum for [Sha] (as well as [hu I138M/I139M]) amyloid. Furthermore, taken together, the NMR data for [hu I138M], [hu I138M/I139M], and [Sha M138I/M139I] amyloids strongly suggest that the M139I mutation is likewise alone sufficient to switch the amyloid core fold of ShaPrP23-144 from [Sha] to [hu]-like.

In order to identify the amino acids that are most critical for converting huPrP23-144 into a [mo]-like amyloid structure, we note that in the immediate proximity of the β-core region hu and mo PrP23-144 differ at only four positions: M109L, M112V, I138M, and S143N (Fig. 1a), with residues 112 and 138 being the only ones that are immobile enough to be detectable in conventional dipolar coupling-based solid-state NMR spectra. Given that the I138M substitution was already demonstrated to have no effect on the structure of [hu] amyloid (Fig. 1b and discussion above), we shifted our attention to residue 112 located at the N-terminal edge of the β-core region for [hu] and [mo] amyloids. In Fig. 1d we compare the ^15^N–^13^Cα NMR spectra recorded for [hu], [mo], and [hu M112V] fibrils (note that [hu M112V/I138M] amyloid was also investigated and found to be virtually identical to [hu M112V]). Although the degree of spectral overlap between [hu M112V] and [mo] amyloids is not as high as that between [hu I139M] and [Sha], it is evident that the M112V mutation alone is sufficient to significantly perturb the [hu] fold and yield fibrils that display many of the spectral (and thus presumably also structural) features of [mo] amyloid, with the residual differences possibly linked to the precise identities of residues 109 and/or 143.

Collectively, the solid-state NMR studies described above indicate that [hu], [mo], and [Sha] fibrils adopt distinct amyloid β-core structures at the atomic level and that these structural differences are largely accounted for by the identities of only two residues (112 and 139) located at the edges of the structured amyloid core region. As summarized in Fig. 1f, the I139M substitution in huPrP23-144 is sufficient to make the protein adopt a [Sha]-like amyloid core structure, while the M112V substitution appears to be primarily responsible for the conversion of huPrP23-144 into a [mo]-like structure.

### Basis for control of core structure by residues 112 and 139

**Fig. 2:**
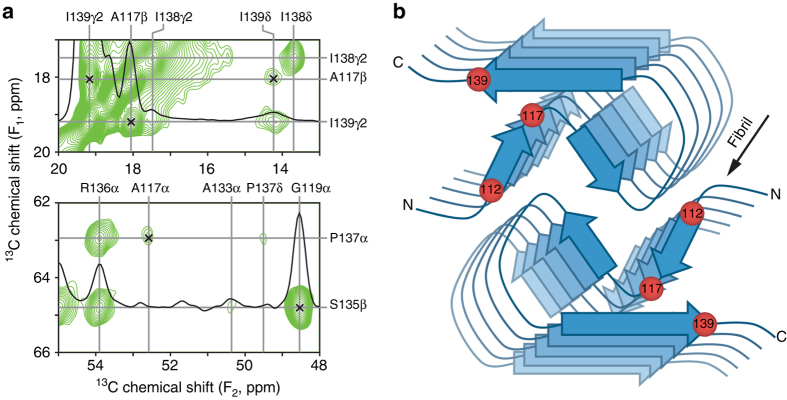
Key interresidue contacts and schematic model of the human PrP23-144 amyloid β-core. **a** Small regions of a 900 MHz two-dimensional ^13^C–^13^C DARR solid-state NMR spectrum recorded with a mixing time of 500 ms for amyloid fibrils generated from huPrP23-144 expressed with 3-^13^C-pyruvate as the carbon source. The spectral regions contain the key restraints on the [hu] amyloid core structure in the form of unambiguous long-range correlations (indicated by *x-marks*) between the following ^13^C atoms: A117Cβ-I139Cγ2, A117Cβ-I139Cδ, A117Cα-P137Cα, and G119Cα-S135Cβ. **b** Schematic model for the [hu] amyloid core based on the combination of solid-state NMR and tilted-beam transmission electron microscopy data (see text for details). In this model [hu] amyloid fibrils consist of two protofilaments in a C_2_-symmetric arrangement with β-sheet regions running parallel to the long fibril axis. The approximate locations of amino acid residues 112, 117, and 139, that have major impact on the structure adopted by PrP23-144 amyloid as discussed in the text, are indicated by *red spheres*

A complete, atomistic understanding of the protein backbone and side-chain interactions involved in stabilizing the amyloid core regions for the different PrP23-144 variants requires the determination of their high-resolution structures—a major undertaking which is currently in progress in our laboratory. In the present study, having established the identity of the amino acid residues that control species-specific differences in the structure of PrP23-144 amyloid, we focus our attention on a rudimentary examination of the structural basis for these differences. In Fig. 2a we show small regions of a 2D ^13^C–^13^C dipolar-assisted rotational resonance (DARR) correlation spectrum^43^, recorded with a long dipolar mixing time (*τ*_mix_ = 500 ms) to highlight key long-range through-space ^13^C–^13^C contacts, for [hu] fibrils generated from huPrP23-144 expressed with 3-^13^C-pyruvate as the sole carbon source^44^. Under the experimental conditions employed in our study the use of 3-^13^C-pyruvate incorporates ^13^C labels at multiple positions, with particularly efficient labeling of Ala-Cβ, Ile-Cγ, Val-Cγ, and Leu-Cδ methyl groups, while reducing the number of strong ^13^C–^13^C dipolar couplings between adjacent ^13^C sites that can lead to dipolar truncation phenomena in uniformly-^13^C-enriched proteins^45^.

The ^13^C–^13^C spectrum displays unambiguous, intense long-range correlations between the side-chains of residues A117 and I139 that are further supported by A117-P137 and G119-S135 couplings (indicated by *x-marks* in Fig. 2a), with additional contacts observed between the side-chain of L125 and residues G114 and A115. Control solid-state NMR experiments performed on diluted fibril samples prepared from a physical mixture of ^13^C,^15^N-labeled, and natural abundance huPrP23-144 in a 1:3 molar ratio (Supplementary Fig. 4) indicate that these correlations are intramolecular, at least in part, with additional possible contributions from neighboring strands in the parallel in-register β-sheet. On the other hand, correlations involving the I138 side-chain are primarily intra-residue or confined to adjacent amino acids. Combined with the MPL data (c.f., Supplementary Fig. 3) and the fact that fingerprint solid-state NMR spectra for [hu] amyloid display a single set of resonances^32, 33^, these prominent long-range contacts have enabled us to propose a schematic structural model for the [hu] fibrils consisting of two protofilaments in a C_2_-symmetric arrangement with β-sheet regions running parallel to the long fibril axis as shown in Fig. 2b. While still largely qualitative, this model is consistent with additional experimental observations reported in our earlier studies^32, 33^. Notably, residues ~115–122, which have the lowest transverse nuclear spin relaxation rates and correspondingly highest signal intensities in dipolar coupling-based solid-state NMR chemical shift correlation spectra and which exhibit the least inhomogeneous line broadening in spectra recorded for frozen hydrated fibril samples, make up a compact, Ala, Gly, and Val-rich hydrophobic core located in the interior of the fibril. On the other hand, residues ~130–139 in the C-terminal β-strand are found at the fibril exterior allowing the side-chain of residue R136, the only charged amino acid present in the [hu] amyloid β-core, to protrude into the solvent. Finally, the structural model readily accommodates the ~90-residue dynamically disordered N-terminal tail by allowing it to extend out of the amyloid core domain with no steric clashes.

The schematic model of [hu] amyloid in Fig. 2b yields several important insights that allow us to rationalize the results of the solid-state NMR analysis for the different PrP23-144 variants. First, the key amyloid structure-determining residues 112 and 139 are located at the edges of the β-core, and in sufficient proximity to allow direct interaction between the amino acid side-chains in this region. Thus, species-specific replacement of either of these residues could act as a “conformational switch” that ultimately impacts the overall structure of PrP23-144 amyloid. Second, the facts that the I139 side-chain points toward A117 in the hydrophobic core and that side-chains of adjacent residues in a β-strand point in opposite directions, indicate that the I138 side-chain points away from the amyloid core—such a topology provides a rationale for why the I138M mutation has no appreciable effect on the structure adopted by [hu] amyloid. Finally, based on the findings that side-chains of residues A117 and I139 are found to be in close spatial proximity and that the relatively conservative I139M substitution has a major effect on the [hu] fibril structure converting it into a [Sha]-like fold, we hypothesize that the side-chains of these two residues in [hu] fibrils engage in specific hydrophobic interactions that stabilize the amyloid β-core and are reinforced by their size complementarity (i.e., a small alanine side-chain interacting with the larger isoleucine).

**Fig. 3:**
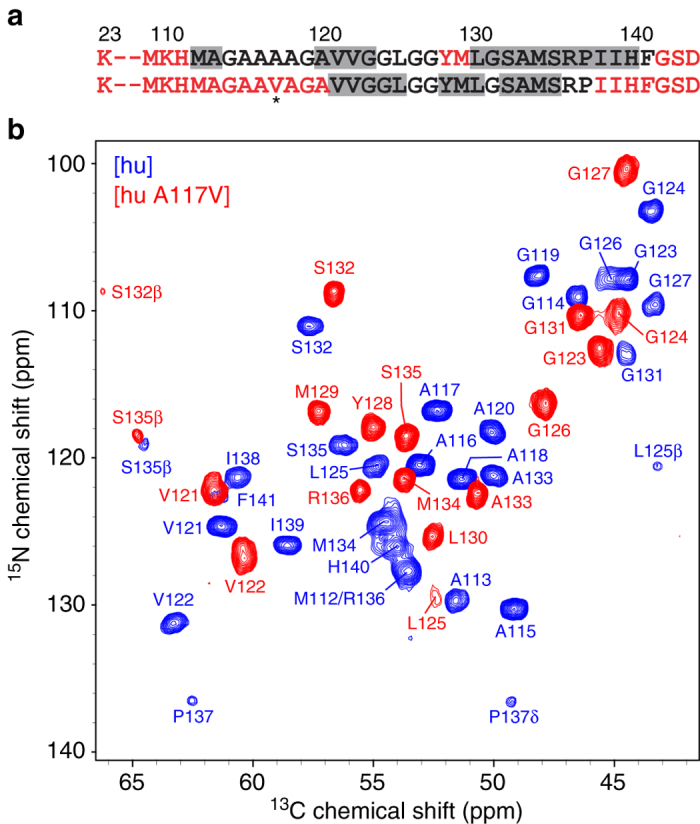
Two-dimensional ^15^N–^13^Cα solid-state NMR spectrum of amyloid fibrils formed by the A117V mutant of human PrP23-144. **a** Amino acid sequences of huPrP23-144 and huPrP23-144 A117V, with the mutation site indicated by an *asterisk*. Immobile residues located within the amyloid core and conformationally flexible residues are shown in *black* and *red fonts*, respectively. Residues with the highest β-strand propensity based on TALOS-N^41^ analysis are indicated by *gray rectangles* (c.f., Fig. 1 and Supplementary Fig. 5). **b** Assigned two-dimensional ^15^N–^13^Cα fingerprint solid-state NMR spectrum of [hu A117V] amyloid fibrils (*red contours*), overlaid with the corresponding spectrum of [hu] amyloid (*blue contours*)

In order to test this hypothesis, we have investigated amyloid formed by the A117V variant of huPrP23-144, which assembles into fibrils with kinetics similar to the wild-type protein as previously reported^46^ but has the small hydrophobic alanine residue replaced by the somewhat larger valine. Based on the solid-state NMR data and structural model in Fig. 2, we expect the putative contacts between the side-chains of residues 117 and 139 that stabilize [hu] amyloid to be perturbed by such an Ala to Val substitution. Indeed, as illustrated in Fig. 3, we find that the fingerprint solid-state NMR spectra of [hu A117V] amyloid differ drastically from those of [hu] amyloid (as well as [mo] and [Sha] amyloids). Based on the sequential resonance assignments established using 2D and 3D solid-state NMR (Fig. 3b) and subsequent TALOS-N secondary structure prediction (Fig. 3a and Supplementary Fig. 5), it appears that the substitution of A117 by the larger valine residue acts to considerably alter the [hu] amyloid β-core structure. Specifically, we find that the rigid core region for [hu A117V] amyloid is shortened relative to [hu], spanning residues ~121–137 (vs. ~112–141 in [hu]) most of which are predicted to adopt a β-strand topology. Most remarkably, signals from residues 117 and 139 are not detected at all in the dipolar coupling-based solid-state NMR spectra of [hu A117V] fibrils suggesting that these residues become more flexible and are no longer part of the rigid amyloid core domain. This finding, which is consistent with the presence of a direct and highly specific interaction between the A117 and I139 side-chains in [hu] amyloid, indicates that when this key interaction is perturbed the PrP23-144 amyloid β-core adopts an alternate thermodynamically stable fold.

### PrP23-144 amyloid polymorphism and new structural strains

**Fig. 4:**
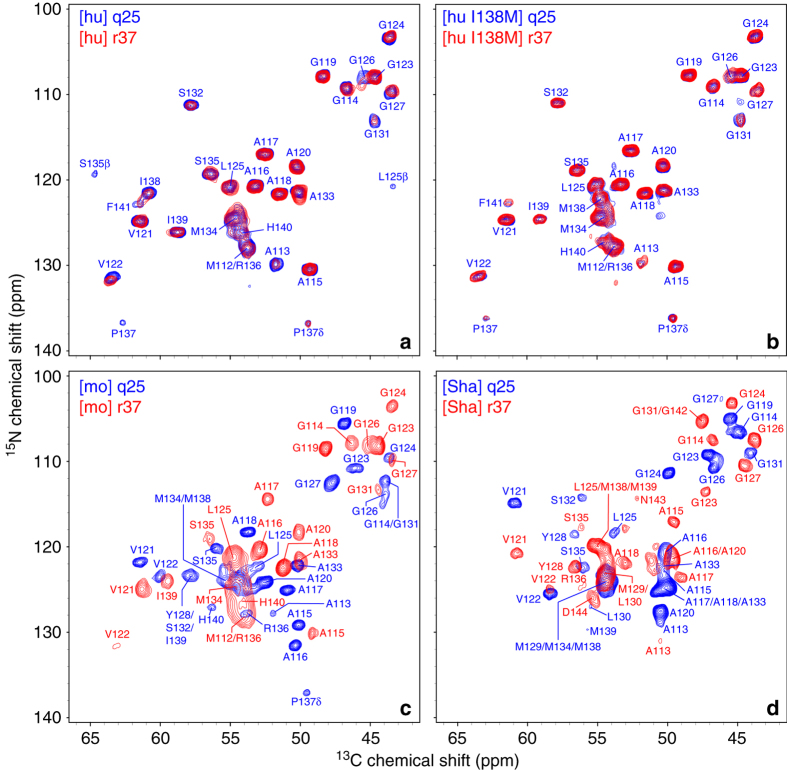
^15^N–^13^Cα solid-state NMR spectra of PrP23-144 fibrils generated at 25 °C under quiescent conditions and at 37 °C with rotation. Assigned two-dimensional ^15^N–^13^Cα fingerprint solid-state NMR spectra are shown for q25 (*blue contours*) and r37 (*red contours*) amyloid fibrils as indicated in the insets for **a** hu, **b** hu I138M, **c** mo, and **d** Sha PrP23-144

Next, we asked the question whether, in addition to being key determinants of species-specific differences in PrP23-144 amyloid structure, residues 112 and 139 could also play a role in molecular level polymorphism and emergence of distinct structural strains of amyloid fibrils. To explore this issue, in addition to fibrils described in the previous sections that were prepared at 25 °C under quiescent conditions (denoted q25), we also examined PrP23-144 fibrils generated at 37 °C with continuous rotation at 8 rpm (denoted r37) as described in the Methods section. Figure 4 compares the fingerprint ^15^N–^13^Cα solid-state NMR spectra for [hu], [hu I138M], [mo], and [Sha] amyloids prepared under these two sets of conditions. These spectra show that [hu] (Fig. 4a) and [hu I138M] (Fig. 4b) amyloids adopt effectively identical core structures for both types of conditions. On the other hand, there are significant chemical shift differences between the spectra of [mo] q25 and [mo] r37 fibrils (Fig. 4c) as well as between the spectra of [Sha] q25 and [Sha] r37 fibrils (Fig. 4d), indicating that structurally distinct amyloid strains can be formed by these proteins depending on the experimental conditions.

Despite clear differences in the fingerprint ^15^N–^13^Cα solid-state NMR spectra, the ^13^C and ^15^N chemical shift assignments indicate that the rigid β-core regions of [mo] and [Sha] strains obtained under the q25 and r37 conditions in each case consist of ~30 C-terminal residues. Furthermore, the [mo] and [Sha] fibrils prepared under both sets of conditions have similar MPL values (Supplementary Fig. 6). Thus, the [mo] and [Sha] amyloid strains generated in the present study have similarly sized core regions and are composed of the same number of protofilaments, with the structural differences between these strains occurring at the level of the folding pattern within the β-core regions.

Both elevated temperature and non-quiescent growth conditions appear to be generally required to access these alternate [mo] and [Sha] core structures. Specifically, for moPrP23-144 the use of continuous rotation at 25 °C or elevated 37 °C temperature in the absence of agitation yields amyloid that is effectively identical to that obtained under the q25 conditions (Supplementary Fig. 7A); only the combination of 37 °C temperature and continuous rotation results in fibrils with distinct fingerprint solid-state NMR spectra. For ShaPrP23-144, the amyloids obtained at either 25 or 37 °C without rotation are virtually indistinguishable, and, while some spectral changes begin to emerge for samples prepared at 25 °C with continuous rotation, the resulting amyloid is clearly distinct from the alternate strain obtained at 37 °C with rotation (Supplementary Fig. 7B).

**Fig. 5:**
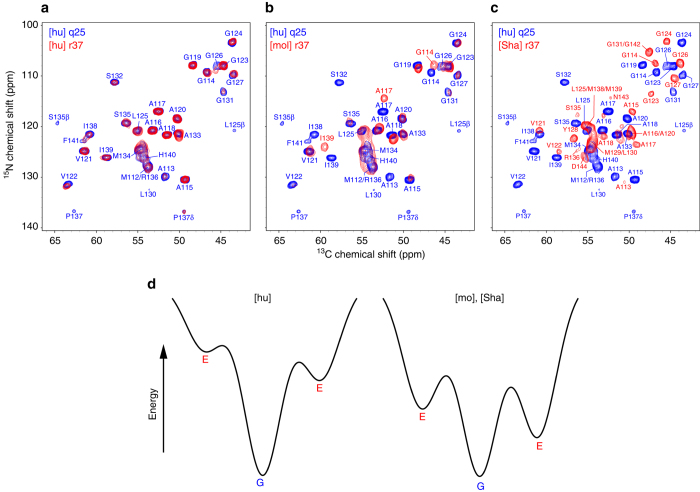
^15^N–^13^Cα solid-state NMR spectra of PrP23-144 fibrils at 37 °C with rotation and energy landscape diagrams for amyloid assembly. **a**–**c** Assigned two-dimensional ^15^N–^13^Cα fingerprint solid-state NMR spectra of **a** [hu], **b** [mo], and **c** [Sha] amyloid fibrils generated at 37 °C with continuous sample rotation at 8 rpm (*red contours*, labeled in the insets as [hu] r37, [mo] r37, and [Sha] r37). Overlaid in each panel is the spectrum of huPrP23-144 fibrils generated at 25 °C under quiescent conditions (q25, *blue contours*). **d** Schematic energy landscape diagrams associated with [hu], [mo], and [Sha] amyloid fibril formation, with the thermodynamically most stable “ground state” and potential alternate “excited state” amyloid folds denoted as “G” and “E”, respectively. The ground state in the energy landscape for each protein in the amyloid state is designated as the structure adopted under quiescent conditions at 25 °C (q25), as under these conditions each of these proteins adopts a unique (presumably lowest free energy) fold which can also be populated for some of the samples even under non-quiescent conditions. Under the specific non-quiescent conditions tested in this study involving rotation at 37 °C (r37), the fold of [hu] amyloid remains unchanged, whereas the mo and Sha proteins are able to adopt alternate amyloid structures through a combination of thermodynamic and kinetic factors. Additional details are provided in the text

Finally, we note that for [mo] and [Sha] amyloids generated at 37 °C with rotation, on occasion, two or more structural polymorphs could be observed within the same macroscopic fibril preparation (Supplementary Fig. 8 for representative data) as opposed to the single, “pure” strains associated with the NMR spectra shown in Figs. 4 and 5. For [Sha] fibrils such polymorphic preparations were typically binary mixtures of the pure q25 and r37 strains (Supplementary Fig. 8A). For some of the [mo] preparations, however, much more complicated fingerprint NMR spectra could be obtained consistent with the presence of mixtures of several amyloid strains (Supplementary Fig. 8B). Most remarkably, however, we find that one of the [mo] structural strains that becomes accessible under the r37 conditions appears to display a three-dimensional fold that is nearly identical to [hu] amyloid at the atomic level, based on the high degree of signal overlap in the ^15^N–^13^Cα fingerprint NMR spectra (Fig. 5b). In contrast, the [Sha] fibrils obtained under the r37 conditions have a β-core fold that is obviously distinct from any of the other PrP23-144 amyloid structures.

As summarized in Fig. 5d, the above findings strongly suggest that, when considered from an energy landscape perspective, the [hu] amyloid core folds into a thermodynamically stable “ground state” structure (represented as “G” in Fig. 5d) over the entire range of experimental aggregation conditions investigated in this study. While huPrP23-144 may conceivably also be able to adopt alternate, “excited state” core structures (denoted by “E” in Fig. 5d), these structures appear to be of sufficiently high energy relative to the ground state fold such that they are clearly not accessible under the conditions explored here. In contrast, the fact that new [mo] and [Sha] polymorphs readily emerge in presence of agitation at elevated temperature (c.f., Fig. 4c, d) points toward far more rugged energy landscapes for these proteins, characterized by the existence of at least one alternate amyloid core structure with energy comparable to the most thermodynamically stable ground state fold. Given the established influence of residues 112 and 139 on the β-core structure adopted by the different PrP23-144 variants under the q25 conditions, it is exceedingly likely that the same amino acids are largely responsible for altering the energy landscapes for mo and ShaPrP23-144 and, through a combination of thermodynamic and kinetic factors, leading to the appearance of new amyloid strains with distinct three-dimensional core folds for these proteins under the r37 conditions.

## Discussion

One of the most puzzling and poorly understood aspects of prion diseases is the phenomenon of interspecies transmissibility barriers. Animal studies and limited experiments in vitro suggest that efficient prion transmission requires “conformational adaptability” of monomeric PrP substrate to the structure of a particular prion aggregate^3, 14^. This model postulates that amino acid sequence variability allows a large spectrum of prion conformations (strains) among different mammalian species, but only a subset of these structures is thermodynamically accessible in a given species (i.e., for a given amino acid sequence). If the structure of a specific donor prion strain is within the range of conformers accessible to PrP of the host, transmission will occur. Conversely, if the structure of the incoming prion strain is incompatible with the spectrum of conformation allowed to the host PrP, this will result in a transmissibility barrier. Even though supported by many observations in vivo and in vitro^3, 14, 26^, this model of transmissibility barriers is largely conceptual in nature, lacking molecular level description. Such description would require detailed structural characterization of prion strains isolated from different species and information regarding the role of specific amino acid residues in formation of these structures. However, high-resolution structural studies with brain-derived prions present major technical challenges that are yet to be overcome.

Previous studies revealed that the phenomena of prion strains and transmissibility (seeding) barriers can be reproduced in vitro using amyloid fibrils formed from the C-terminally truncated PrP variant PrP23-144^25, 26^. Even though the latter system cannot recapitulate the full complexity of prion propagation in vivo, the mechanistic insight provided by these studies is conceptually consistent with the picture emerging from animal experiments. This, together with amenability of PrP23-144 amyloid fibrils to high-resolution structural characterization by solid-state NMR spectroscopy^32–36^ and recent finding that these fibrils are infectious^10^, suggests that this system may be of unique value for exploring mechanistic principles of transmissibility barriers at the structural level.

In the present study, we have used solid-state NMR to gain the initial high-resolution insight into the nature of the structural differences between PrP23-144 amyloid fibrils corresponding to three different species: human, mouse, and Syrian hamster. Previous low-resolution studies using atomic force microscopy and infrared spectroscopy concluded that structural differences between [hu] and [Sha] are controlled by residues 138 and 139^26^, both of which are occupied by Ile in huPrP and Met in ShaPrP. However, the present high-resolution experiments conclusively demonstrate that the sole determinant of these structural differences is the nature of the amino acid at position 139, as species-specific substitution of this residue is sufficient to convert the solid-state NMR spectrum of [hu] amyloid to that characteristic of [Sha] amyloid. Analysis of the solid-state NMR data also reveals that under quiescent fibril growth conditions different structures are adopted at the atomic level by [hu] and [mo] amyloids. No such differences could be detected in previous low-resolution studies. Akin to the [hu] and [Sha] pair, the structural differences between [hu] and [mo] amyloids appear to be largely controlled by a single amino acid residue. In this case, however, the critical residue is at position 112, which in huPrP is occupied by Met and in moPrP by Val (both proteins have Ile at position 139). No spectral differences were observed upon species-specific Ile to Met substitution at position 138 in [hu] amyloid, further confirming that the identity of residue 138 is of little consequence for the structure of PrP23-144 amyloid fibrils.

The crucial role of residues 112 and 139 as determinants of species-dependent structural differences may be rationalized by inspection of the solid-state NMR-based schematic model of a parallel in-register β-structure for [hu] amyloid (Fig. 2b). As described in detail in the Results section, individual monomers fold in such a way that the M112 and I139 side-chains, located at opposite ends of the amyloid β-core, point toward the interior of this core and are in sufficient proximity to each other and additional hydrophobic side-chains to allow direct interactions. Thus, species-specific replacement of either of these two residues can act as a conformational switch, impacting the overall structure of PrP23-144 amyloid. On the other hand, the side-chain of I138 points away from the β-core and is not involved in any direct interactions with other side-chains. This may explain why species-dependent substitution of Ile 138 with Met has no effect on the structure of [hu] amyloid.

Even though caution should be exercised when extrapolating observations in a model system to a situation in vivo, our finding that residue 139 is one of key determinants of species-dependent structural differences between PrP23-144 amyloid fibrils is in line with previous reports that the nature of the residue at this position (or its equivalent) is a major determinant of prion transmissibility barriers between different species^22, 47^. On the other hand, the present observation that the Ile to Met replacement at position 138 is of little consequence for the structure of PrP23-144 amyloid is intriguing in light of previous observations that the same amino acid replacement has a major impact on the kinetics of amyloid formation, greatly affecting the lag phase^25^. The latter finding suggests that the side-chain of residue 138 may be directly involved in intermolecular and/or intramolecular interactions that lead to the formation of an aggregation nucleus that is likely oligomeric in nature. However, these early interactions do not appear to be preserved in the final amyloid structure, as in the latter the side-chain of residue 138 is not in contact with other side-chains. In contrast, the identity of the amino acid residue at position 112 impacts the final structure of PrP23-144 amyloid, but—according to previous data^25^—not the kinetics of amyloid formation. Thus, it appears that there is no direct link between the effect of individual amino acid residues in PrP23-144 on the nucleation process and the impact of these residues on the final amyloid β-core structure. In a pathobiological context, nucleation is believed to play a key role in prion formation de novo in sporadic prion diseases, whereas the specific structure of prion aggregates is a key determinant of transmissibility barriers.

Perhaps even more intriguing is the finding that amyloids formed under identical quiescent conditions by human and mouse PrP23-144 are structurally distinct, while no seeding barriers are observed under these conditions between proteins from the two species (i.e., [hu] amyloid can seed fibrillization of moPrP23-144 and [mo] amyloid can seed amyloid formation by huPrP23-144)^25, 26^. In the context of the previously proposed conformational adaptability model^26^, such cross-seeding compatibility would suggest that the structure of [hu] amyloid is within the spectrum of structures that can be adopted by moPrP23-144, and the structure of [mo] is within the spectrum of structures that are accessible to huPrP23-144. The potential for such conformational adaptability is supported by our observations that, even though structurally distinct at the atomic level, [hu] and [mo] amyloids formed under the q25 conditions exhibit similar secondary structures consistent with similar overall β-core folds. Furthermore, our data indicate that under the r37 conditions moPrP23-144 can assemble into several structurally distinct amyloid strains, one of which appears to be effectively identical to [hu] amyloid. Structural polymorphism appears to be a general property of amyloids formed by many different proteins (for a recent review see ref. ^48^). Furthermore, recent data indicate that prions, which often exist as a pool of multiple strains, may undergo evolutionary adaptation to the environment, a process that can potentially lead to selection of a particular prion strain that replicates most efficiently in a given environment^49, 50^. Further studies are needed to determine whether such a selection would occur in reactions seeded with structurally heterogeneous population of [mo] r37 amyloid.

An alternative possibility is that cross-seeding could result in “strain switching”, a poorly understood phenomenon observed for both yeast and mammalian prions^3, 14, 51–53^. One hypothetical mechanism that could lead to such strain switching proposes that adaptation of newly recruited monomer to the structure of the seed takes place only within the portion of the β-core, whereas outside this critical region conformational features may arise from inherent (sequence-based) preferences rather than from templating by the seed^46^. The present findings provide a stepping stone for rigorous testing of these different possibilities by systematically analyzing the structures of PrP23-144 amyloids generated in cross-seeding reactions. Furthermore, even though additional amino acid residues (outside of the 23-144 region of PrP) are also known to play a role in species barriers for prion transmissibility, mechanistic insight into cross-seeding barriers in the experimentally accessible PrP23-144 amyloid model system provides a conceptual framework for future studies with infectious aggregates formed by the full-length PrP. However, high-resolution solid-state NMR experiments with the latter systems are at present hampered by factors such as insufficient quantities and heterogeneity of highly infectious material that can be generated from recombinant full-length PrP by currently available methods.

## Methods

### Protein expression and purification

Plasmids encoding human, mouse, and Syrian hamster PrP23-144, with N-terminal linkers containing a His_6_-tag and thrombin cleavage site, were described previously^25, 54^. Constructs for the expression of hu M112V, A117V, I138M, I139M, M112V/I138M, I138M/I139M, and Sha M138I/M139I PrP23-144 were generated by site-directed mutagenesis using primers listed in Supplementary Table 1 and the QuikChange II site-directed mutagenesis protocol (Stratagene), and confirmed by sequencing of the entire genes (Genewiz). Isotopically ^13^C,^15^N enriched PrP23-144 variants were expressed in *Escherichia coli* BL21 (DE3) cells using M9 minimal media containing ^15^NH_4_Cl (1 g L^−1^) and ^13^C-glucose, 1,3-^13^C-glycerol (2 g L^−1^), 2-^13^C-glycerol (2 g L^−1^), or 3-^13^C-pyruvate (3 g L^−1^) as the nitrogen and carbon sources, respectively, and purified by nickel affinity chromatography using Ni-NTA resin (Qiagen), followed by cleavage of the N-terminal His_6_-tag using biotinylated thrombin (Novagen), sequestration of the thrombin using streptavidin-agarose beads (Novagen), and removal of the residual His_6_-tag by dialysis against ultrapure water^32, 36, 54^.

### PrP23-144 amyloid fibrils for solid-state NMR analysis

Amyloid fibrils were prepared by adding 1 M potassium phosphate pH 6.4 buffer to a final concentration of 50 mM, to a 400 μM (~5 mg mL^−1^) solution of PrP23-144 in ultrapure water^36^. The majority of PrP23-144 fibril samples in this study were generated at 25 °C under quiescent conditions (denoted q25), where the fibril suspensions were incubated largely unperturbed except for gentle inversion of the sample tubes every 12 h to ensure complete mixing^36^. In addition, several amyloid fibril samples were prepared at 37 °C under quiescent conditions (denoted q37) and at 25 or 37 °C with continuous rotation at 8 rpm (denoted r25 and r37, respectively), as indicated in the Results and Discussion sections. Note that, depending on the PrP23-144 sequence, the quantitative conversion of monomers into mature fibrils, as assessed by TEM and monitoring of the UV spectra for the supernatant following sample centrifugation, was typically complete within ~6–24 h under the r37 conditions versus ~2–7 days under the q25 conditions. Atomic force microscopy was routinely used to characterize the resulting PrP23-144 amyloid fibrils^26, 36^, which were subsequently washed with the 50 mM potassium phosphate pH 6.4 buffer and transferred by centrifugation to 3.2 mm solid-state NMR zirconia rotors^36^.

### Solid-state NMR spectroscopy

NMR data for sequential resonance assignments were collected using a 500 MHz Varian VNMRS spectrometer equipped with a 3.2 mm BioMAS ^1^H–^13^C–^15^N probe and an 800 MHz Bruker Avance III HD spectrometer equipped with a 3.2 mm Efree ^1^H–^13^C–^15^N probe. The ^13^C and ^15^N resonance assignments were established as described in our previous studies^32, 36^, using a set of 2D and 3D chemical shift correlation spectra including ^15^N–^13^Cα, ^15^N–^13^Cα–^13^CX, ^15^N–^13^C΄–^13^CX, and ^13^C΄–^15^N–^13^Cα. The 2D ^13^C–^13^C DARR^43^ correlation spectrum with mixing time of 500 ms was recorded at the National High Magnetic Field Laboratory (NHMFL) on a 900 MHz ultrawide bore instrument^55^ outfitted with a Bruker Avance III console and a 3.2 mm sensitivity-enhanced Low-E probe designed and built at NHMFL^56, 57^. All NMR experiments were carried out at magic angle spinning frequency of 11.111 kHz and effective sample temperature of 5 °C. NMR data were processed in NMRPipe^58^ and analyzed in Sparky^59^ and nmrglue^60^.

### Data availability

Further data and data sets generated during and/or analyzed during the current study are available from the corresponding author on reasonable request.

## Electronic supplementary material

Supplementary Information
