# Supplementary material for: Species-dependent structural polymorphism of Y145Stop prion protein amyloid revealed by solid-state NMR spectroscopy
Source: Nat Commun. 2017 Sep 29;8:753. doi: 10.1038/s41467-017-00794-z (PMC5622040; doi:10.1038/s41467-017-00794-z)
Supplement: Supplementary file 1 — Supplementary Information [file 41467_2017_794_MOESM1_ESM.pdf]

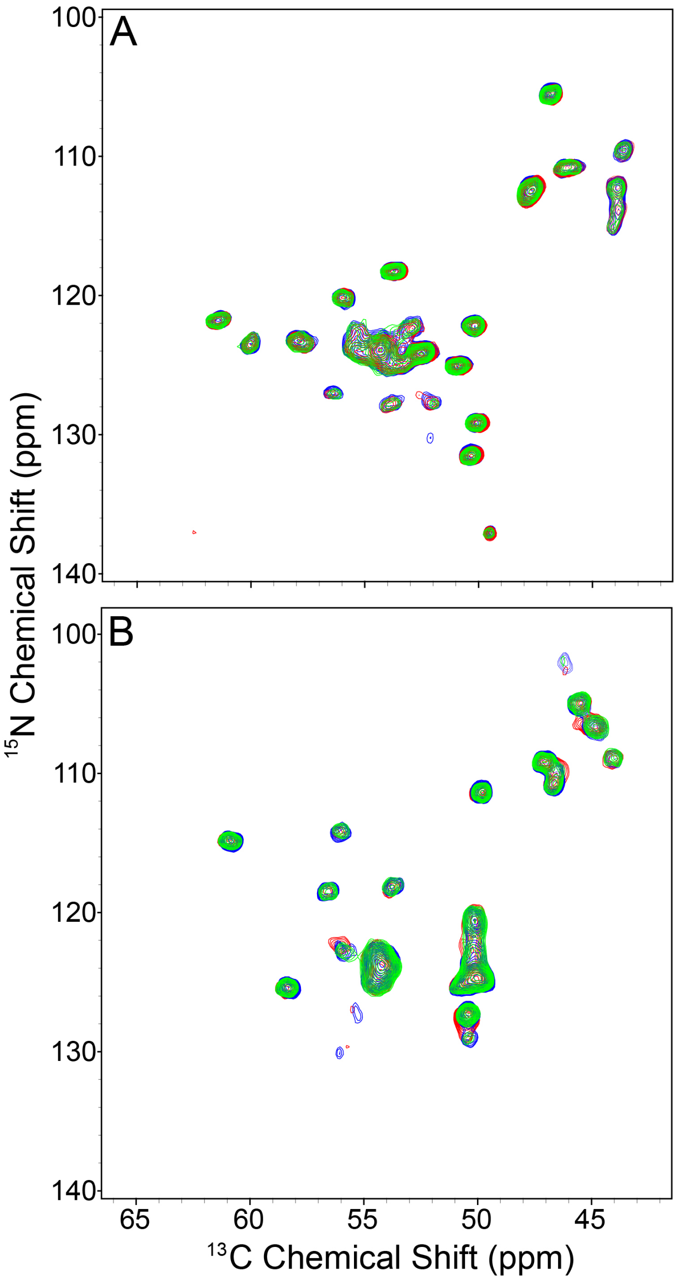

Supplementary Figure 1. Comparison of two-dimensional ^{15}N-$^{13}C\alpha$ solid-state NMR spectra recorded for multiple independent preparations of (**A**) [mo] and (**B**) [Sha] fibrils under quiescent conditions at 25 ^{o}C. The fact that for each protein the spectra are virtually superimposable illustrates the high degree of reproducibility of PrP23-144 fibrils generated using this method.

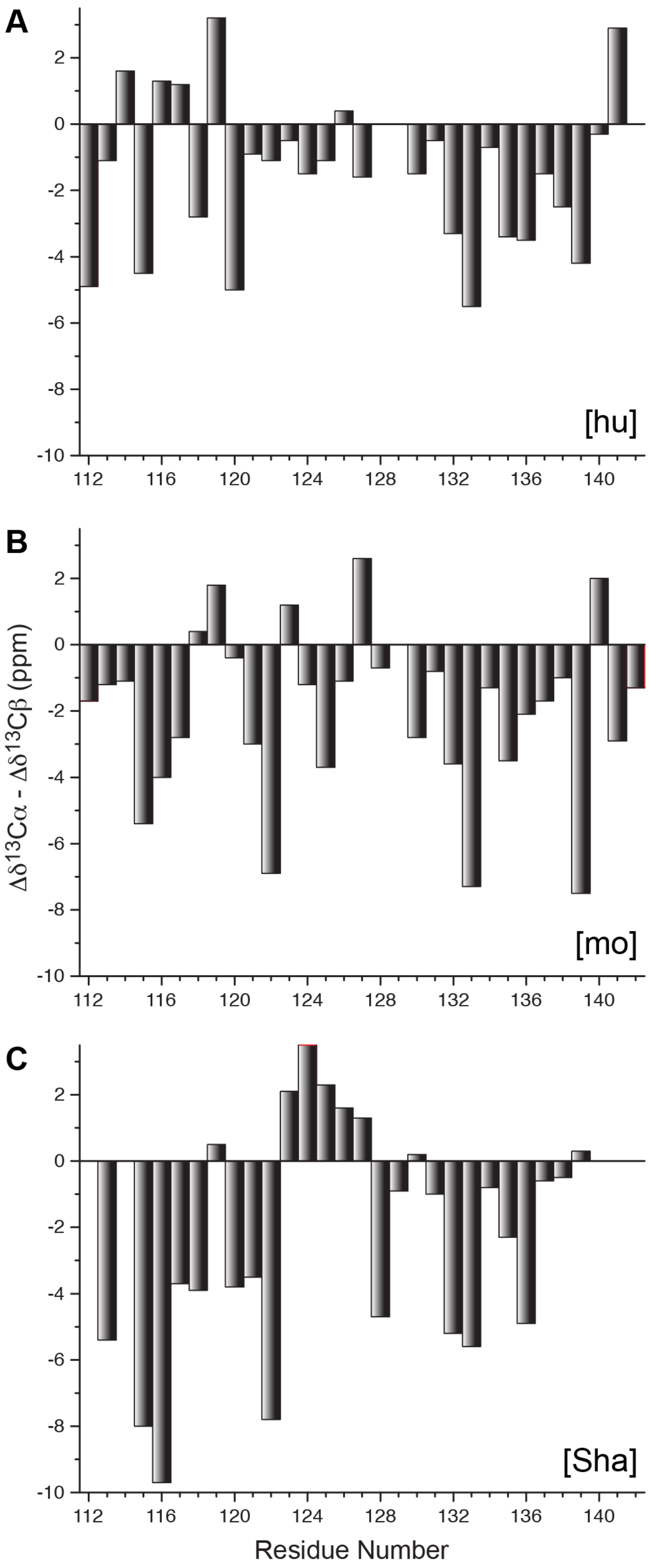

Supplementary Figure 2. Plots of $^{13}C\alpha$ - $^{13}C\beta$ secondary chemical shift differences ($\Delta\delta^{13}C\alpha$ - $\Delta\delta\,^{13}C\beta$) for (**A**) [hu], (**B**) [mo] and (**C**) [Sha] amyloid fibrils prepared at 25 ^{o}C under quiescent conditions.

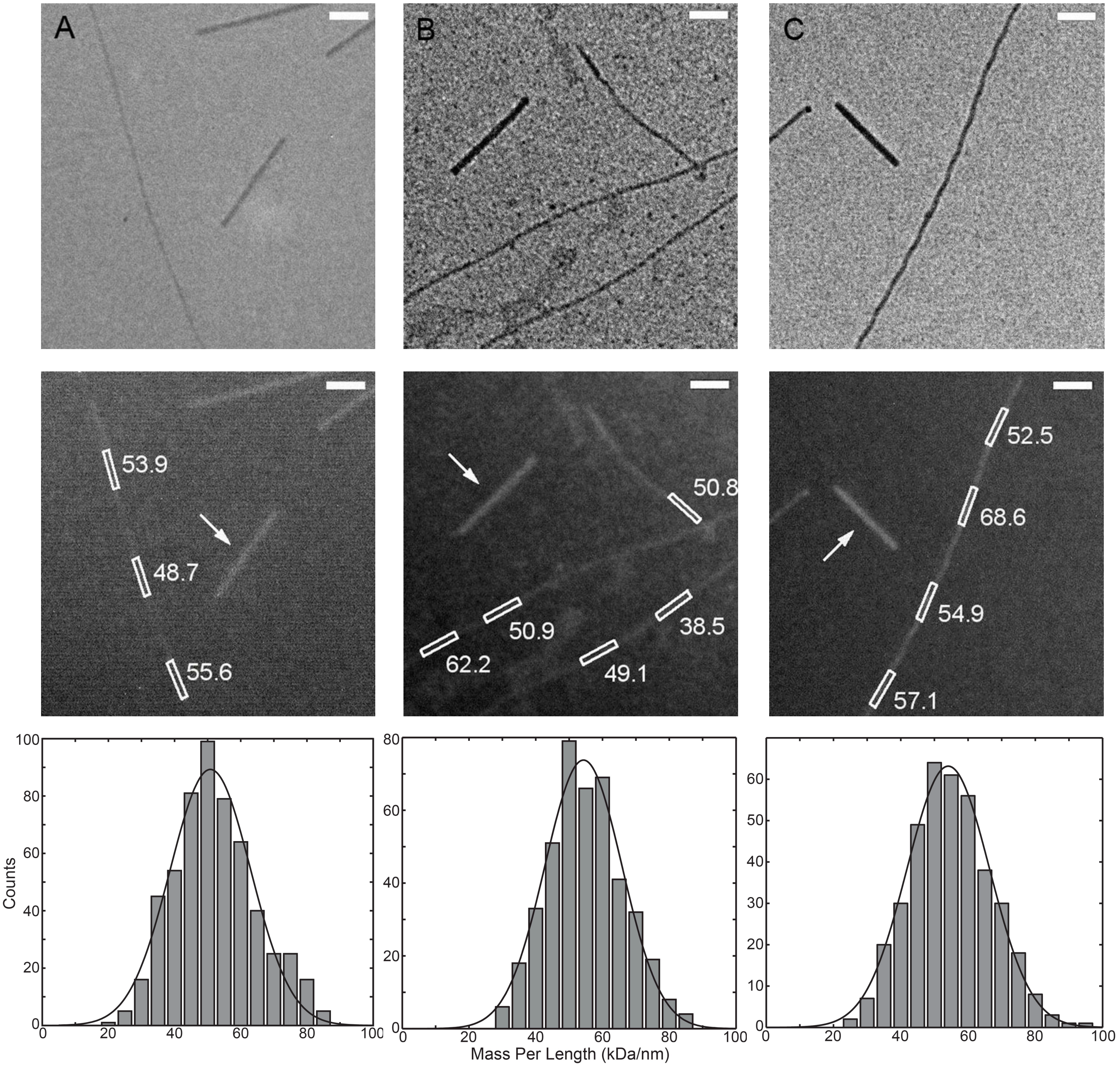

Supplementary Figure 3. Bright-field TEM images of unstained amyloid fibrils (top), and the corresponding dark-field TB-TEM[1] images (middle) and mass-per-length (MPL) histograms (bottom) for (**A**) huPrP23-144, (**B**) moPrP23-144 and (**C**) ShaPrP23-144. The fibril samples were prepared at 25 ^{o}C under quiescent conditions. Representative MPL values determined for multiple amyloid fibril segments are shown in the TB-TEM images, and the locations of reference tobacco mosaic virus particles are indicated by arrows. The scale bars in all TEM images correspond to 100 nm. The MPL histograms were each constructed using ca. 400-600 counts and fit to Gaussian functions. The average MPL values were found to be 50.8 ± 1.2 kDa/nm for [hu] fibrils, 54.3 ± 0.9 kDa/nm for [mo] fibrils and 54.1 ± 0.6 kDa/nm for [Sha] fibrils.

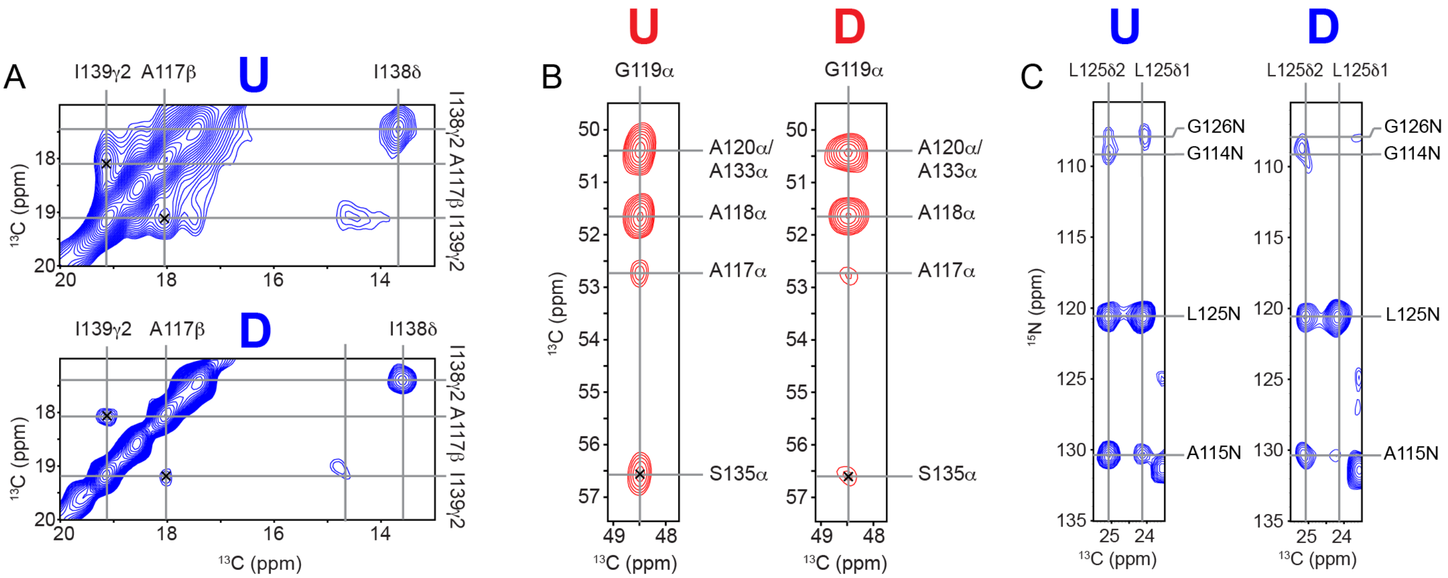

Supplementary Figure 4. Small regions of (A,B) ^{13}C-^{13}C DARR (τ_{mix} = 500 ms) and (C) ^{15}N-^{13}C TEDOR[2] (τ_{mix} = 18 ms) solid-state NMR spectra for undiluted fibrils prepared from ^{13}C,^{15}N-labeled huPrP23-144 (marked U) and diluted fibrils (marked D) prepared from a physical mixture of ^{13}C,^{15}N-labeled huPrP23-144 and unlabeled huPrP23-144 in a 1:3 molar ratio. ^{13}C,^{15}N-huPrP23-144 used to generate the fibril samples for these experiments was prepared using labeling schemes based on 1,3-^{13}C-glycerol (panels (A) and (C)) and 2-^{13}C-glycerol (panel (B)).[3] The comparison of the undiluted and diluted fibril spectra shows that correlations between residues 117 and 139, 119 and 135, and 114/115 and 125 are intramolecular, at least in part, with additional possible contributions from neighboring strands in the parallel in-register β-sheet, as opposed to arising from contacts between protofilaments.

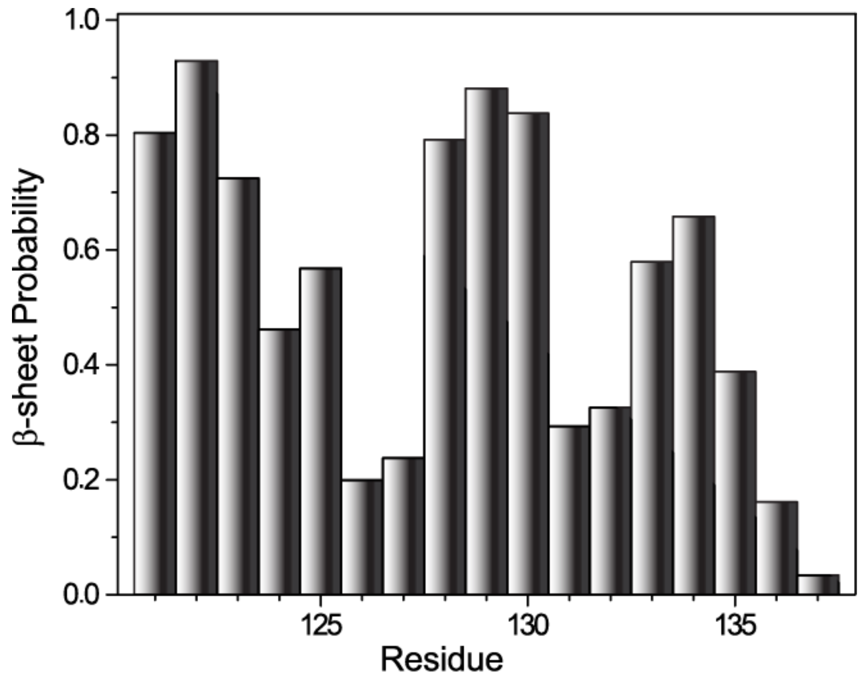

Supplementary Figure 5. Residue specific β-sheet probability for [hu A117V] amyloid fibrils predicted by TALOS-N.[4]

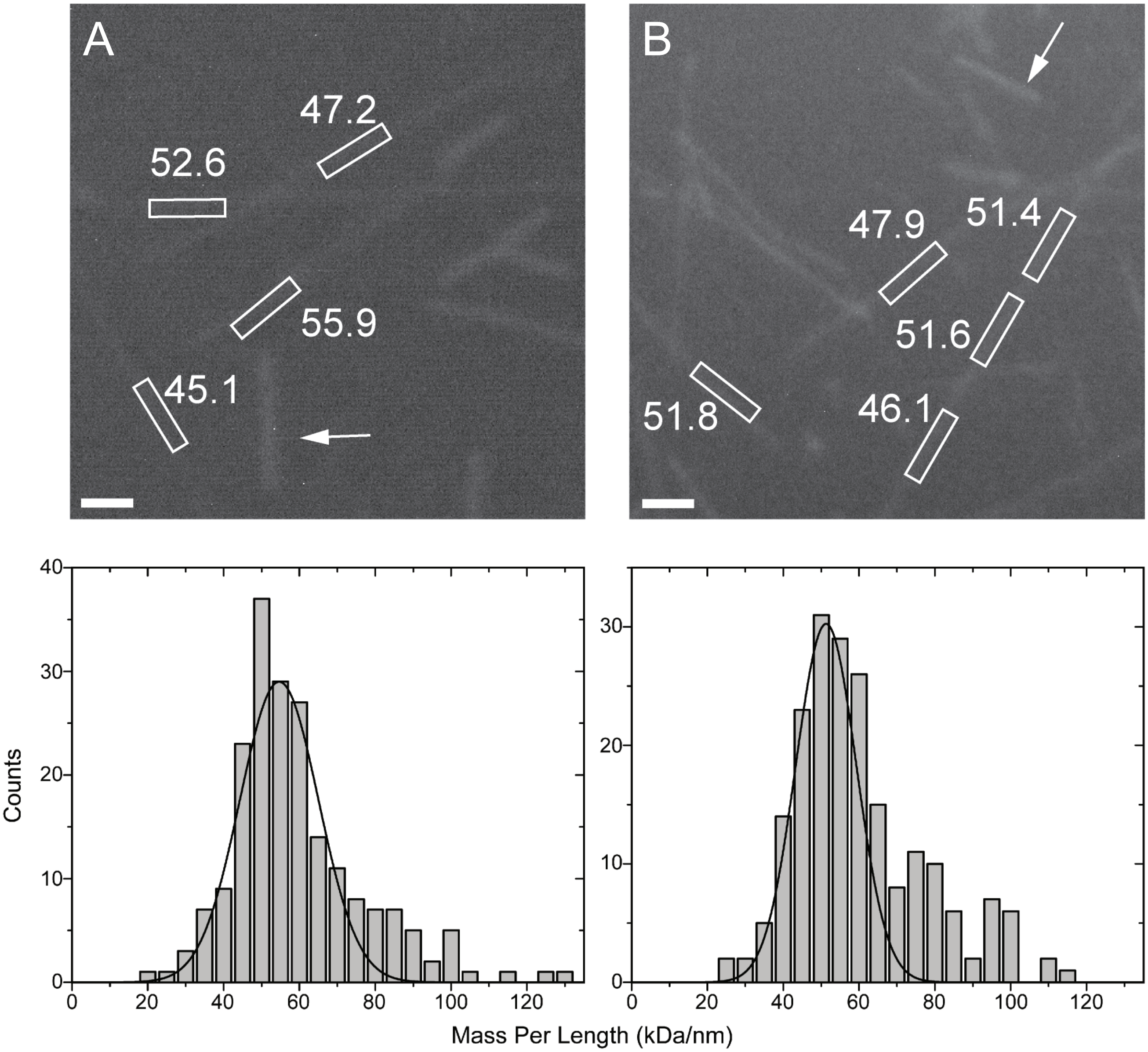

Supplementary Figure 6. Dark-field TB-TEM images (top) and corresponding mass-per-length histograms (bottom) for (**A**) moPrP23-144 and (**B**) ShaPrP23-144 amyloid fibrils prepared at 37 ^{o}C with continuous rotation at 8 rpm. Representative MPL values are shown in the TB-TEM images, and the locations of reference tobacco mosaic virus particles are indicated by arrows. The scale bars correspond to 100 nm. Note that fibrils obtained under the r37 growth conditions were considerably shorter in length relative to those generated using the q25 method (c.f., Supplementary Figure 3), and, consequently, the MPL measurements for these samples were somewhat complicated by the macroscopic aggregation and co-localization of these shorter fibril fragments on the EM grid surface. The MPL histograms were each constructed using 200 counts, and found to consist of a major component at around 40-60 kDa/nm MPL and a smaller population of fibrils with apparently higher MPL values of ~80-120 kDa/nm (likely corresponding to multiple fibrils). The histograms were fit to Gaussian functions to yield average MPL values of 54.6 ± 1.4 kDa/nm for individual [mo] fibrils and 51.3 ± 1.1 kDa/nm for the [Sha] fibrils.

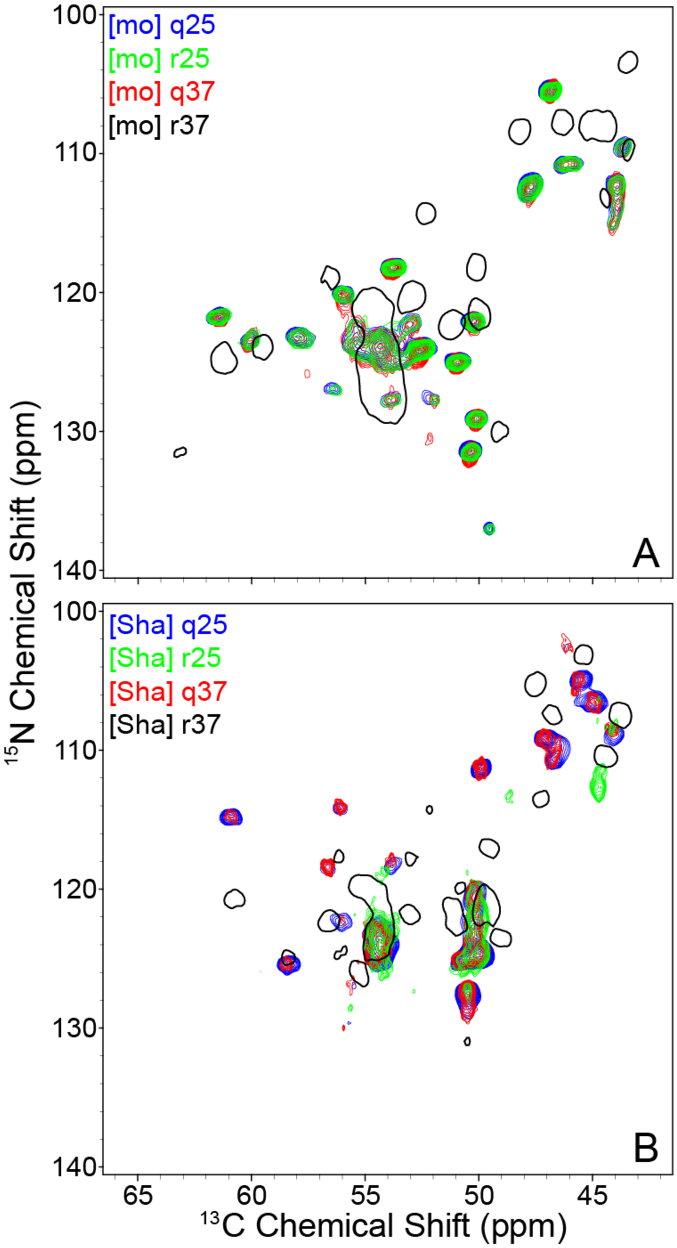

Supplementary Figure 7. Effect of elevated temperature and continuous sample rotation on the conformation of (**A**) [mo] and (**B**) [Sha] amyloid fibrils. For both [mo] and [Sha] fibril samples, the ^{15}N-^{13}Cα fingerprint solid-state NMR spectra spectra are denoted as follows: q25 = 25 ºC, quiescent (blue contours), r25 = 25 ºC, continuous sample rotation at 8 rpm (green contours), q37 = 37 ºC, quiescent (red contours), r37 = 37 ºC, continuous sample rotation at 8 rpm (single black contour). These spectra show that both elevated temperature and continuous sample rotation are generally required to access the alternate [mo] and [Sha] amyloid structures discussed in the text. In contrast, the structure of the amyloid β-core for huPrP23-144 fibrils is invariant over the entire range of experimental aggregation conditions investigated in this study (c.f., Figure 4).

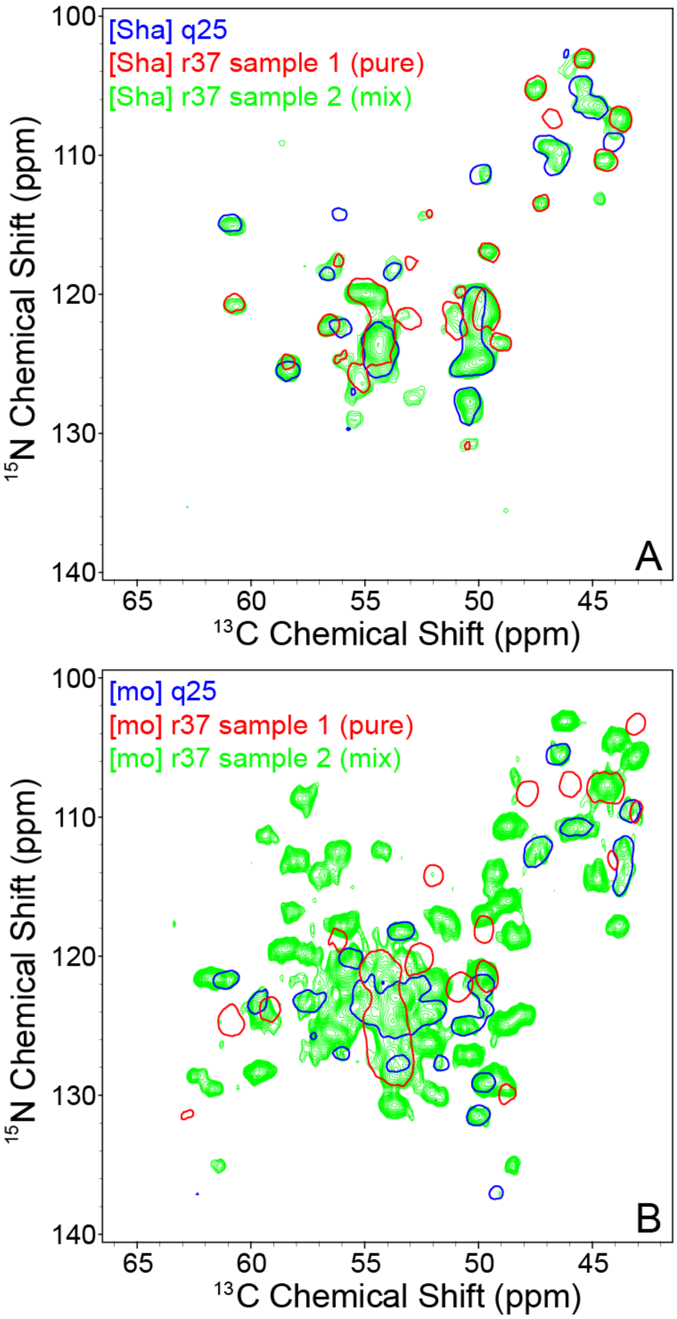

Supplementary Figure 8. ^{15}N-^{13}Cα solid-state NMR spectra representative of [Sha] (top) and [mo] (bottom) fibrils consisting of mixutres of multiple fibril polymorphs (green contours), observed on occasion for the [mo] and [Sha] amyloid samples prepared at 37 °C with continuous rotation as noted in the text. For reference, the corresponding NMR spectra of [mo] and [Sha] fibrils grown under quiescent conditions at 25 ºC are shown as single blue contours and spectra of pure [mo] and [Sha] strains generated at 37 ºC with continuous rotation and discussed in the text (c.f., Figure 4 and Figure 5) are shown as single red contours.

Supplementary Table 1. Primers used to generate PrP23-144 mutants by site-directed mutagenesis.

Plasmid	Forward primer sequence (5'-3')	Reverse primer sequence (5'-3')
hu M112V	CCAACATGAAGCACGTGGCTGGTGCTGCAGCAG	CTGCTGCAGCACCAGCCACGTGCTTCATGTTGG
hu A117V	CTGGTGCTGCAGTAGCTGGGGCAG	CTGCCCCAGCTACTGCAGCACCAG
hu I138M	ATGAGCAGGCCCATGATCCATTTCGGC	GCCGAAATGGATCATGGGCCTGCTCAT
hu I139M	GCCATGAGCAGGCCCATCATGCATTTCGGCAGTGAC	GTCACTGCCGAAATGCATGATGGGCCTGCTCATGGC
hu I138M/I139M	AGCAGGCCCATGATGCATTTCGGCAGTG	CACTGCCGAAATGCATCATGGGCCTGCT
Sha M138I/M139I	GAGCAGGCCCATCATACATTTTGGC	GCCAAAATGTATGATGGGCCTGCTC

*The hu M112V/I138M plasmid was generated in two steps using the primers for the M112V and I138M mutations.

Supplementary References

1 Chen, B., Thurber, K. R., Shewmaker, F., Wickner, R. B. & Tycko, R. Measurement of amyloid fibril mass-per-length by tilted-beam transmission electron microscopy. *Proc. Natl. Acad. Sci. USA* **106**, 14339-14344 (2009).

2 Jaroniec, C. P., Filip, C. & Griffin, R. G. 3D TEDOR NMR experiments for the simultaneous measurement of multiple carbon-nitrogen distances in uniformly ^{13}C,^{15}N-labeled solids. *J. Am. Chem. Soc.* **124**, 10728-10742 (2002).

3 Castellani, F. *et al.* Structure of a protein determined by solid-state magic-angle spinning NMR spectroscopy. *Nature* **420**, 98-102 (2002).

4 Shen, Y. & Bax, A. Protein backbone and sidechain torsion angles predicted from NMR chemical shifts using artificial neural networks. *J. Biomol. NMR* **56**, 227-241 (2013).
